# Clozapine Safety in Pregnancy: A Clinical Study

**DOI:** 10.1093/schbul/sbae132

**Published:** 2024-07-20

**Authors:** Jayashri Kulkarni, Adam De Chellis, Heather Gilbert, Emmy Gavrilidis, Eveline Mu, Leila Karimi, Qi Li

**Affiliations:** HER Centre Australia, Department of Psychiatry, School of Translational Medicine, Monash University, Melbourne, Australia; HER Centre Australia, Department of Psychiatry, School of Translational Medicine, Monash University, Melbourne, Australia; HER Centre Australia, Department of Psychiatry, School of Translational Medicine, Monash University, Melbourne, Australia; HER Centre Australia, Department of Psychiatry, School of Translational Medicine, Monash University, Melbourne, Australia; HER Centre Australia, Department of Psychiatry, School of Translational Medicine, Monash University, Melbourne, Australia; HER Centre Australia, Department of Psychiatry, School of Translational Medicine, Monash University, Melbourne, Australia; HER Centre Australia, Department of Psychiatry, School of Translational Medicine, Monash University, Melbourne, Australia

**Keywords:** clozapine, quetiapine, antipsychotic medication, prenatal, postnatal, gestational diabetes

## Abstract

**Background and Hypothesis:**

Pregnant women with persistent schizophrenia and related disorders may require ongoing antipsychotic treatment, including clozapine. However, the potential risks of using clozapine during pregnancy and the postnatal period remain uncertain.

**Study Design:**

We conducted a nested case-control study using the National Register of Antipsychotic Medication in Pregnancy (NRAMP) database. Our study assessed pregnancy outcomes among Australian women diagnosed with schizophrenia spectrum disorder and treated with clozapine (*n* = 14) during the first trimester. These women were compared to 2 subgroups: those treated with quetiapine (*n* = 53) and those not taking any medication (*n* = 24) during pregnancy.

**Study Results:**

We observed higher rates of miscarriage in the clozapine group compared to the quetiapine and drug-free groups. The clozapine group had a higher early pregnancy body mass index but lower overall pregnancy weight gain than the other groups. The prevalence of gestational diabetes was significantly higher in the clozapine group. The percentage of vaginal delivery was higher in the clozapine group than in the other 2 groups. Neonatal outcomes such as gestational age, and Apgar scores were similar across groups. The birth weight was lower in the clozapine group compared to the other 2 groups.

**Conclusions:**

This study suggests that pregnant women taking clozapine and their babies have greater adverse outcomes compared to other groups. Clozapine appears to be associated with a greater risk of miscarriages, maternal gestational diabetes, and lower birth weight. However, the gestational age, Apgar scores, and admission to NICU/SCN were comparable between all groups.

## Introduction

Antipsychotics are effective in treating schizophrenia and other primary psychosis disorders.^1^ Clozapine is regarded as the treatment for persistent psychosis and is commonly prescribed for individuals with schizophrenia who have not responded to 2 different antipsychotic medications.^2,3^ While the adverse effects of clozapine are well documented,^4^ its specific impact during pregnancy and the postnatal period remains largely unknown.^5^

Vigod et al. found that antipsychotic use during pregnancy does not independently raise the risk of adverse maternal and perinatal outcomes compared to non-users.^6^ Comprehensive systematic reviews^7,8^ also suggest that exposure to second-generation antipsychotics like aripiprazole, olanzapine, and quetiapine in utero is not linked to higher risks of major congenital malformations. However, Damkier and Videbech^8^ reviewed 23 studies covering 14 382 pregnant women using second-generation antipsychotics, noting limited safety data for clozapine during pregnancy. Specifically, the review lacked sufficient information on outcomes like miscarriage, stillbirth, small for gestational age infants, and neurodevelopment in neonates and children.

Several studies on antipsychotic medication during pregnancy suggest a weak association with increased risk of congenital malformations,^9,10^ cardiac issues, and respiratory distress in the neonate,^11,12^ low birth weight,^13^ poor neonatal adaptation,^14^ and maternal gestational diabetes.^15^ A Danish study reported a higher risk of miscarriage in pregnancies exposed to quetiapine,^16^ although confounding factors like smoking, alcohol, illicit substance use, and diet were not thoroughly considered.

Data on the association between neonatal gestational age at birth or birth weight and prenatal antipsychotic exposure are scarce. Bodén et al. did not observe any statistical differences in neonatal gestational age at birth or birth weight in 187 infants exposed to in utero clozapine or olanzapine, compared with unexposed infants or infants exposed to other antipsychotic medications.^15^ In contrast, Newham et al. found that infants exposed to clozapine or olanzapine (*n* = 25) in utero weighed significantly more than infants exposed to first-generation antipsychotics (*n* = 45) but not unexposed infants (*n* = 38).^17^ These studies subsumed clozapine and olanzapine under the 1 exposure category, thereby lacking specificity.

Peng et al. found no significant differences in Apgar scores at 1 or 5 min in 33 infants exposed to clozapine during pregnancy compared with unexposed infants.^18^ However, data were reported as aggregated for all second-generation antipsychotics. Newport et al. found no significant difference between infants exposed to quetiapine compared with infants exposed to other antipsychotic medications in utero, which, as noted above, did not include clozapine.^19^

Due to ethical imperatives, including beneficence and non-maleficence-based obligations toward pregnant women and their unborn offspring, none of the studies related to antipsychotic medication use during pregnancy meet the gold standard of randomized, placebo-controlled, double-blind, cross-research. Consequently, most of the available evidence regarding clozapine’s safety in pregnancy has been derived from observational^8^ or case studies.^20^ However, these were inherently limited in supporting causal inferences, and many were characterized by methodological shortcomings that compounded the problem.

The treatment of pregnant women with antipsychotic-resistant psychosis poses a significant ethical dilemma.^21^ Without clozapine, these women may experience severe and potentially life-threatening psychotic episodes. However, clozapine use during pregnancy is associated with potential maternal and fetal risks. Clozapine can increase maternal glucose levels, leading to gestational diabetes, which can adversely affect fetal development. Additionally, the sedative effects of clozapine might result in reduced fetal movement and other developmental concerns. This leaves clinicians with the challenging task of balancing the potential harm to the fetus against the severe consequences of untreated or inadequately treated psychosis in the mother. Ongoing research and a better understanding of the impacts of antipsychotic use during pregnancy are needed. Our study was an analysis of a subset of data from our prospective cohort study (2005–19), called the National Register of Antipsychotic Medication in Pregnancy (NRAMP) (ClinicalTrials.gov identifier NCT00686946). Previous findings from earlier analyses of NRAMP data have been published^12,22,23^ and these prior publications detail the NRAMP methodology. The study we now present concerns data from 3 groups of NRAMP participants who took quetiapine, clozapine, or no antipsychotic drugs for at least the first trimester of pregnancy. Among all NRAMP participants enrolled, quetiapine was the most commonly prescribed antipsychotic, a trend noted in other studies.^24,25^ By examining the maternal and newborn outcomes for the 3 pregnant groups, that is those treated with clozapine, quetiapine, and no antipsychotic medication at least during the first trimester; we hope that our study will enrich the small evidence base currently available regarding clozapine’s safety in pregnancy.

## Methods

### Study Design and Population

Our clozapine study is nested in our larger NRAMP (ClinicalTrials.gov NCT00686946) study. The NRAMP study comprised women prescribed first or second-generation antipsychotic drugs who were enrolled in the observational study that followed women through their pregnancy and then their babies for the first 12 months of life. The NRAMP study enrolled women from the study’s inception in 2005 to a cutoff date for data analysis on March 31, 2019.

The inclusion criteria for enrollment into NRAMP were:

• Women taking antipsychotic medication during pregnancy
• Women who were pregnant or had a baby in the last 12 months
• Women living in Australia
• Women who were able to provide informed consent

The exclusion criteria were the opposite of the above inclusion criteria.

Women were referred from sites including general practice clinics, public and private hospitals, community mental health centers, private practitioners, and universities. Once referred and assessed as suitable for participation, women were then followed up prospectively. Throughout the woman’s involvement in the study, her treating team provided ongoing care, including medication and monitoring. This study received ethics approval from the Alfred Hospital Ethics Committee (Ethics approval number 114/04).

In this clozapine study, the exposure group included women from the NRAMP database who had a diagnosis of a schizophrenia spectrum disorder and took clozapine or quetiapine (at minimum) during the first trimester of pregnancy. The non-exposed/control group were also women with a diagnosis of schizophrenia spectrum disorder who chose not to take any antipsychotic during the first trimester of pregnancy (at a minimum).

### Primary Outcomes

The primary outcomes include pregnancy outcomes (live birth or miscarriages), delivery mode (vaginal or cesarean), gestational diabetes mellitus (GDM) status, and weight gain.

### Secondary Outcomes

The secondary outcomes are the neonatal outcomes including baby anomalies (fetal/neonatal respiratory distress), gestation age at birth, birth weight, Apgar scores, and critical care/special care requirements.

### Statistical Analysis

Variables used in this study included baby birth status (livebirth or not), delivery model (vaginal or cesarean), GDM status (presence or absence), maternal overall weight gain from pregnancy to baby birth, baby respiratory distress, SCN (Special Care Nursery) or NICU (Neonatal Intensive Care Unit) admission, Apgar score (1 and 5 min), birth weight, and baby gestation age. These variables were divided into 4 groups: maternal categorical, maternal continuous, neonatal categorical, and neonatal continuous (Table 1).

**Table 1.** Description of Outcome Variables in This Study

Variable Group	Variable Types	Variable Illustration
Obstetric	Categorical	• Birth status (livebirth or not) • Delivery model (vaginal or cesarean) • GDM status (presence or absence)
	Continuous	• Overall pregnancy weight gain (units: kilogram)
Neonatal	Categorical	• Respiratory distress (presence or absence) • SCN or NICU admission (required or not required) • APGAR scores 1 min • APGAR scores 5 min
	Continuous	• Birth weight (units: kilogram) • Gestation age (units: week)

The demographic and clinical characteristics are reported through descriptive statistics. Shapiro test was used to evaluate data distribution. Where sample size allowed, the Kruskal-Wallis H test and ANOVA were used to evaluate group differences in characteristics and outcome variables for continuous and dichotomous variables. Analysis of complete case analysis as the primary analysis was used for this study without imputation for missing data, given the impact of the missing data was negligible.^26^ The confounding variables were analyzed using univariate models with just 1 explanatory variable at a time. Family history of diabetes was used as a covariate when analyzing GDM. All the demographic variables with significant differences between groups were used as covariates in the regression analysis. Sensitivity analysis was performed for all confounding variables, and twins were included in this analysis as individuals. All the analyses were performed through IBM SPSS (Version 27) and R (Version 4.2.3).

## Results

We analyzed data from a total of 24 women who decided not to take antipsychotic drugs during the first trimester, 14 participants taking clozapine, and 53 participants taking quetiapine, all prescribed during the first trimester. Table 2 summarizes the baseline demographic and clinical characteristics of these participants, collected at study entry and during the first trimester (between 8 and 12 weeks).

**Table 2.** Baseline Demographic-Maternal Characteristics of the No-drug, Clozapine, and Quetiapine at First Trimester

	No-drug (*N* = 24)	Clozapine (*N* = 14)	Quetiapine (*N* = 53)	*P*
Age, years (Mean ± SD)	31.33 ± 3.24	34.07 ± 5.64	31.51 ± 4.96	.155
Early pregnancy BMI (Mean ± SD)	*N* = 24	*N* = 11	*N* = 47	
All^a^	25.55 ± 4.85	32.36 ± 5.94	28.65 ± 6.93	.008
BMI ≥25	29.08 ± 4.05	33.3 ± 5.70	31.98 ± 5.86	.140
BMI <25	21.33 ± 1.07	23.00 ± 0	21.55 ± 2.45	.560
Marital status^a^				.023
Married	15/24 (62.5%)	2/13(15.38%)	24/52 (46.15%)	
Single	9/24 (37.5%)	11/13(84.62%)	28/52 (5.85%)	
Educational attainment				.122
Primary/secondary education	5/23 (21.7%)	3/10 (30.00%)	11/50 (22.0%)	
Tertiary education	8/23 (34.8%)	5/10 (50.00%)	23/50 (46.0%)	
Postgraduate education	10/23 (43.5%)	2/10 (20.00%)	16/50 (32.0%)	
Smoking (Yes, %)	14/24 (58.3%)	10/13 (76.9%)	34/50 (68.0%)	.453
Smoking per day during pregnancy (Yes)				Not applicable
<5	2	2	7	
6–10	1	0	7	
11–20	0	0	1	
>20	1	0	0	
Missing	0	6	3	
Alcohol consumption (Yes, %)	3/24 (12.5%)	4/12 (33.3%)	11/49 (22.4%)	.341
Illicit substance use (Yes, %)	2/24 (8.3%)	2/13 (15.4%)	1/49 (2.0%)	.094
Mood stabilizers use (Yes)				Not applicable
Sodium valproate	3	0	1	
Lithium	3	1	3	
Tegretol	0	0	6	
Missing	1	6	3	
Folate use during pregnancy (Yes, %)	18/21 (85.1%)	10/10 (100%)	45/49 (91.8%)	.298
Family history of diabetes (Yes, %)	12/24 (50.0%)	8/12 (66.7%)	25/51 (49.0%)	.515
Antidepressant first trimester^a^ (Yes, %)	6/24 (25.0%)	8/12 (66.7%)	28/53 (52.8%)	.026

Data are *n*/*N* (%). BMI, body mass index; *N*, participants number; SD, standard deviation.

^a^Significant difference; Significant level: *P* < .05.

All participants in the 3 groups had schizophrenia spectrum disorder as their primary psychiatric diagnosis. Significant differences were found in early pregnancy BMI, marital status, and use of antidepressants in the first trimester. However, no significant difference was found in BMI subgroups, either larger or less than 25. Compared to participants exposed to quetiapine or no drugs, those exposed to clozapine were less likely to be married or in a de facto relationship. Clozapine-exposed participants also had a greater BMI in early pregnancy and increased illicit substance use, smoking, and alcohol consumption during pregnancy compared to the other groups. Additionally, a majority of clozapine-exposed participants had a family history of diabetes (66.7%).

### Obstetric Outcomes

**Fig. 1.**
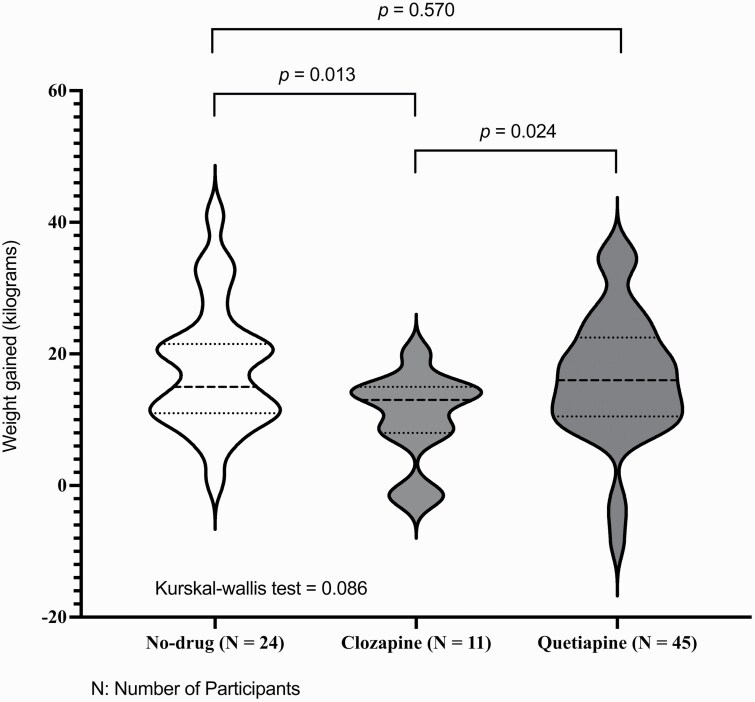
Comparison of weight gained from pre-pregnancy to baby delivery between 3 groups.

The main obstetric outcomes are presented in Table 3 and Figure 1. Pregnancy outcomes (livebirth vs miscarriage/stillbirth) were significantly different between clozapine-exposed vs no-drug (*P* = .043) and clozapine-exposed vs quetiapine-exposed groups (*P* = .033). We only had the gestational age of 1 reported miscarriage in the clozapine group, which was a late-term miscarriage at 37 weeks. Weight gained was significantly lower among clozapine vs quetiapine and no-drug groups despite having a family history of diabetes as presented in Figure 1. Diagnosis of GDM was over 70% in the clozapine group, which was significantly higher than the no-drug group (8.3%) and the quetiapine group (23.9%) (*P* < .001 for no-drug and *P* = .002 for the quetiapine groups separately).

**Table 3.** Obstetric Outcomes of No-drug, Clozapine, and Quetiapine

	No-drug (*N* = 24)	Clozapine (*N* = 14)	Quetiapine (*N* = 53)	*P*
Pregnancy outcome				Not applicable
Livebirth	24/24 (100%)	11/14 (78.6%)	47/48 (97.9%)	
Miscarriage	0/24 (0%)	3/14 (21.4%)	1/48 (2.1%)	
Delivery mode				.194
Vaginal delivery	14/23 (60.9%)	8/11 (72.7%)	20/44 (45.5%)	
Cesarean delivery	9/23 (39.1%)	3/11 (27.3%)	24/44 (54.5%)	
GDM^a^				*<*.001
Yes	2/24 (8.3%)	8/11 (72.7%)	11/46 (23.9%)	

Data are *n*/*N* (%). BMI, body mass index; GDM, gestational diabetes mellitus; *N*, participants number; SD, standard deviation.

^a^Significant level: *P* < .05.

### Neonatal Outcomes

**Fig. 2.**
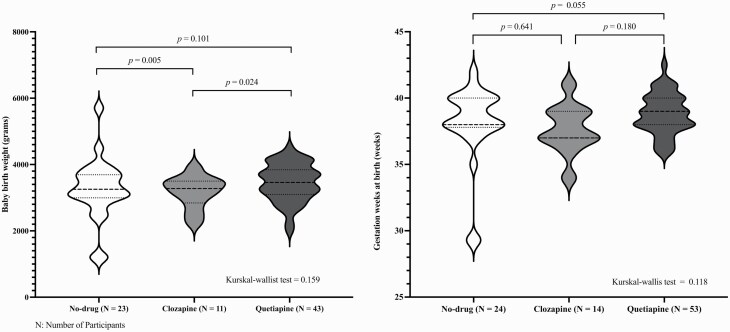
Comparison of baby birth weight and gestation weeks at birth between 3 groups.

The neonatal outcomes including baby anomalies (fetal/neonatal respiratory distress), gestation age at birth, birth weight, Apgar scores, and critical care/special care requirements are presented in Table 4. As shown, fetal/neonatal respiratory distress was higher among quetiapine (40.91%), followed by the clozapine group (27.3%) than the no-drug (0%). Gestation age appeared to be relatively similar between groups ranging from around 37 to 38 weeks without any significant differences as displayed in Figure 2. Although the clozapine group had over 70% admission rate to an SCN/NICU, which is greater than the no-drug [odds ratio: 4.15; 95% CI 0.864–19.92; *P* = .076] and quetiapine group [odds ratio: 3.20; 95% CI 0.748–13.691; *P* = .117], these differences were not significant. The baby’s birth weight was significantly lower in the clozapine-exposed babies than in the other groups, and birth weight was significantly higher in the quetiapine-exposed group compared to the clozapine-exposed group.

**Table 4.** Neonatal Outcomes of No-drug, Clozapine, and Quetiapine

	No-drug (*N* = 24)	Clozapine (*N* = 14)	Quetiapine (*N* = 53)	*P*
Fetal/neonatal respiratory distress				Not applicable
Yes	0/24 (0%)	3/11 (27.3%)	18/44 (40.91%)	
Gestational age at birth (weeks, Mean ± SD)	38.39 ± 2.40	37.64 ± 1.77	38.82 ± 1.54 (*N* = 44)	.118
Birth weight (grams, Mean ± SD)	*N* = 23	*N* = 11	*N* = 43	.159
	3317.21 ± 804.29	3177.27 ± 458.76	3452.60 ± 561.48	
Apgar score (Mean ± SD)	*N* = 21	*N* = 8	*N* = 40	
1 min^a^	8.33 ± 0.94	7.75 ± 1.71	8.08 ± 1.49	.867
5 min^a^	8.93 ± 0.39	9.00 ± 0	8.99 ± 0	.556
NICU/SCN admission				.171
Yes	9/23 (39.1%)	8/11 (72.7%)	20/44 (45.5%)	
No	14/23 (60.9%)	3/11 (27.3%)	24/44 (54.5%)	

Data are *n*/*N* (%); NICU, neonatal intensive care unit; SCN, Special care nursery; SD, standard deviation.

^a^Significant difference; Significant level: *P* < .05.

### Group Comparisons

The results of sensitivity analysis adjusted for BMI, marital status, antidepressant use in the first trimester, smoking, drinking, and illicit drug use during pregnancy through a logistic regression model are summarized in Table 5, and a significant difference was found in the diagnosis rate of GDM among the 3 groups.

**Table 5.** Regression Results of Neonatal and Obstetric Outcomes

Variable	B	SE	*P*	z	OR	95% CI
Obstetric						
Delivery model	−0.038	0.036	.300	−1.044	0.963	0.907–1.022
Weight gain overall	−0.010	0.008	.218	−1.242	0.990	0.978–1.003
GDM	0.474	0.140	.001	3.381	1.606	1.276–2.023
Neonatal						
Feta or respiratory distress	−0.411	0.322	.206	−1.275	0.663	0.390–1.127
Gestational age at birth	0.014	0.033	.660	0.442	1.015	0.961–1.072
Baby birth weight	0.000	0.000	.958	−0.053	1.000	1.000–1.000
Apgar score—1 min	−0.031	0.059	.601	−0.525	0.969	0.878–1.069
Apgar score—5 min	0.160	0.152	.297	1.052	1.173	0.914–1.507
NICU or SCN admission	0.017	0.156	.910	0.113	1.018	0.787–1.317

GDM, gestational diabetes mellitus; NICU, neonatal intensive care unit; SCN, special care nursery.

B: beta; SE: standard error; z: z score; OR: odds ratio; CI: confidence interval; GDM: gestational diabetes mellitus; NICU: neonatal intensive care unit; SCN: special care nursery

There were no significant differences found in the sensitivity analysis as displayed in Supplementary Tables 1–4.

## Discussion

This study is an important prospective, observational study to evaluate the pregnancy outcomes of a cohort of women specifically taking clozapine. The results yield some important preliminary insights of clinical relevance into the possible effects of clozapine on the obstetric and neonatal outcomes of pregnant women and their babies.

### Obstetric Outcomes

Among the main obstetric outcomes included in the analysis, pregnancy outcomes (livebirth vs miscarriage/stillbirth) were significantly different between clozapine and no-drug and quetiapine groups. The early pregnancy BMI was significantly higher in the clozapine group compared to the quetiapine and no-drug group. Overall weight gain was lower in the clozapine group, although these contrasts (clozapine vs no-drug; clozapine vs quetiapine) were not statistically significant. It is crucial to consider that the clozapine-exposed group had a higher early pregnancy BMI, indicating preexisting obesity. This study, therefore, examines not only the risks associated with clozapine use but also the compounded risks that come with obesity during pregnancy.

Miscarriage, particularly in the context of antipsychotic use, is a crucial outcome to consider. Our results indicate that the risk of miscarriage was higher in the clozapine-exposed group compared to the no-drug and quetiapine group. The impact of miscarriage can vary depending on the gestational age at which it occurs. In the current study, we only had the gestational age information for 1 clozapine group participant who suffered a miscarriage at 37 weeks, and understandably experienced huge grief and sadness. Future studies should ensure this information is collected for all participants to understand better and address the varied impacts of miscarriage in the context of antipsychotic use.

The risk of developing GDM in the clozapine group was significantly higher than in the other groups. Over 70% of clozapine subjects were diagnosed with GDM, which was over 9 times the incidence of GDM in the no-drug group, over 3 times the quetiapine group in this study, and over 4 times the general population.^27^ GDM is associated with an increased risk of women developing preeclampsia, type 2 diabetes, metabolic syndrome, and cardiovascular disease.^27,28^ High gestational age and macrosomia are the most common adverse neonatal outcomes associated with GDM, with macrosomia, in turn, being associated with an increased risk of operative delivery and secondary complications.^27^ However, the clozapine group in our study had no higher gestational age and lower birth weight than the other groups. Nonetheless, mental health, obstetric, and midwifery clinicians should be aware of the potential for increased risk of GDM in women taking clozapine and to collaborate in minimizing this risk through early targeted screening and preventive interventions.

### Neonatal Outcomes

Gestational age, birth weight, and Apgar scores were reasonably comparative between the groups. Notably, over twenty percent of all clozapine-exposed babies showed signs of fetal or respiratory distress compared to zero percent in the no-drug group. In the quetiapine group, over 40% of babies experienced fetal/respiratory distress, compared to 72% of the clozapine-exposed babies. Clozapine’s role in these adverse events needs to be interpreted with caution in light of the small sample size and effects of potential confounders.

Over 70% of all clozapine-exposed babies were admitted to an SCN or NICU, which was 4 times the rate in the general Australian population.^29^ This finding serves as a reminder to clinicians treating pregnant women to consider all the risks of prescribing clozapine since this medication impacts significantly on the developing fetus and subsequent baby. Unfortunately, if the woman has persistent and severe psychosis, with poor response to other antipsychotics but good response to clozapine; then clozapine treatment will be needed during pregnancy. It is important to maintain the woman’s mental health during pregnancy and beyond, since her function as a mother is critical for the newborn’s development, as well as her right to enjoy her time as a new mother. Furthermore, it is important for pregnant women taking clozapine or quetiapine to have access to SCN, NICU, and excellent obstetric and pediatric services to ensure safe outcomes for both the woman and her newborn.

### Limitations

This study has a number of significant limitations. For some subjects (as displayed in Tables 2–4), information regarding particular outcomes was missing. This would have had the effect of reducing statistical power, thereby increasing the probability of failing to detect a significant between-group difference. It might also have biased parameter estimates, depending on the nature of missingness. Short of missing data points, the source data was lacking in descriptive detail.

Several variables were identified as potential confounders based on between-group comparisons and examination of individual cases. These included early pregnancy BMI and polypharmacy. A larger sample size would invite more robust and detailed model specifications (ie, controlling for confounders) that would minimize the potential for biased estimates.

Arguably this study’s most significant limitation was its small sample size and, therefore, low power; thus, the findings need to be interpreted with caution. Notionally, the easiest way to increase power is to increase the sample size. However, in a study such as this 1, where subjects comprising the population of interest (pregnant women with a psychotic disorder who give consent) were scarce. This is challenging. Thus, despite its limitations, this study serves an important exploratory function in highlighting key areas for further research and some important clinical pointers. Future research to inform clinical guidelines would benefit from recruiting a much larger sample, with the capacity to balance key confounders, and, therefore, provide more robust findings.

### Clinical Considerations

Treating pregnant women with antipsychotic-resistant psychosis presents a significant challenge for clinicians. Without the use of clozapine, these women risk experiencing severe and potentially life-threatening psychotic episodes. Alternatives such as quetiapine, brexpiprazole, cariprazine, and other newer antipsychotics may be considered, but they often do not provide the same level of symptom control for patients who have not responded to other treatments, and there are limited data on these newer antipsychotics in terms of safety in pregnancy. Clinical judgement is required to determine the true need for clozapine treatment in pregnant women. If clozapine is required to maintain the woman’s mental health, then her treating team will need to ensure that the best possible tertiary hospital obstetrics and pediatric services are available for her antenatal care, safe delivery of her baby, and postnatal mental health monitoring. The risks and benefits of taking clozapine during pregnancy should be discussed with the woman and her nominated caregivers, to promote a better understanding of the need for good antenatal care and excellent obstetric involvement in the birthing process. Extra and early monitoring for fetal developmental issues and maternal metabolic disturbances are required. It is also important for clinicians to assist the pregnant patient to establish and maintain stable mental health throughout the pregnancy and beyond, in order to maximize the woman’s enjoyment and participation in mothering her newborn.

Addressing the clinical dilemma about continuing to prescribe clozapine during pregnancy requires a nuanced approach involving individualized treatment plans and close monitoring of maternal and fetal health. The decision to use clozapine during pregnancy is a difficult balancing act where all potential risks and benefits for the woman and her developing baby need to be considered. The lack of equally effective alternatives for some patients with treatment-resistant psychosis highlights the need for ongoing research and development of safer antipsychotic options for use during pregnancy.

## Conclusion

As the current literature attests, women of childbearing age with schizophrenia spectrum disorders are at increased risk of a number of adverse pregnancy outcomes. It is also important to note that clinically, clozapine is currently the “persistent psychosis” medication and often is used as the last option after many other antipsychotics have been trialed. Hence people prescribed clozapine tend to have more severe psychoses. As is generally the case for drugs that pose more than minimal risks to pregnant women and their unborn children, our current understanding of clozapine’s reproductive safety has only emerged piecemeal through observational research efforts. Thus, in the absence of randomized controlled trials (ethically not viable), there is an ongoing need for lower-level study designs, with large samples paired with sophisticated and considered statistical analysis approaches, to bring our knowledge closer approximation to clozapine’s real-world effects on pregnancy outcomes. Addressing limitations of prior studies, this study represents such an effort and offers some informative albeit tentative insights, which are consistent with previous studies. Compared to no-drug and quetiapine, clozapine might be associated with a greater risk of certain adverse outcomes for both mother and baby, including miscarriages, GDM, and lower birth weight. We found that the neonatal outcomes, including gestational age at birth, Apgar score, and NICU/SCN admission, were not statistically different between groups. Of course, this study is subject to significant limitations. Nonetheless, it highlights areas for further research, ideally using larger sample sizes and comparing individual antipsychotics with adjustment for relevant confounders, exploration of mediating mechanisms of the clozapine and potential adverse outcome relationships, and the need for examining the impact of clozapine use throughout the pregnancy. Information derived from such studies would greatly help women, and their doctors make more informed decisions about the benefits versus risks associated with different antipsychotic choices pre-pregnancy and thereby contribute to optimal pregnancy outcomes.

## Supplementary Material

sbae132_suppl_Supplementary_Tables
