# Supplementary material for: Clozapine Safety in Pregnancy: A Clinical Study
Source: Schizophr Bull. 2024 Jul 20;52(2):sbae132. doi: 10.1093/schbul/sbae132 (PMC12996916; doi:10.1093/schbul/sbae132)
Supplement: sbae132_suppl_Supplementary_Tables [file sbae132_suppl_supplementary_tables.docx]

**Supplementary Table 1.** Sensitivity analysis in the subgroup (Marital Status = Yes)

|  |  | Control | Clozapine | Quetiapine |  |
| --- | --- | --- | --- | --- | --- |
| Birth Mode |  |  |  |  | *p* = 0.997 |
|  | Live birth | 15 | 2 | 23 |  |
|  | Miscarriage | 0 | 0 | 0 |  |
|  | Missing | 0 | 0 | 1 |  |
| Delivery Model |  |  |  |  | *p* = 0.880; OR = 1.043 [0.654,1.667] |
|  | Vaginal | 7 | 1 | 6 |  |
|  | Vaginal/Forceps | 1 | 1 | 0 |  |
|  | Vaginal/Venteuse | 0 | 0 | 1 |  |
|  | Vaginal/Venteuse and Forceps | 1 | 0 | 0 |  |
|  | Emergency LSCS | 4 | 0 | 5 |  |
|  | Elective LSCS | 1 | 0 | 9 |  |
|  | Missing | 1 | 0 | 3 |  |
| Weight gain overall (kilogram) |  | 18.34+10.00 | 6.5+12.02 | 16.42 + 6.15 | *p* = 0.703; OR =1.033 [0.895,1.203] |
| GDM |  |  |  |  | *p* = 0.637; OR= 1.045 [0.891,1.243] |
|  | **Without family history** |  |  |  |  |
|  | No | 8 | 1 | 10 |  |
|  | Yes | 1 | 0 | 0 |  |
|  | **With family history** |  |  |  |  |
|  | No | 6 | 1 | 7 |  |
|  | Yes | 0 | 0 | 7 |  |
| Fetal distress |  |  |  |  | *Not applicable* |
|  | No | 14 | 2 | 22 |  |
|  | Yes | 1 | 0 | 2 |  |
| Gestational age (weeks) |  | 38.69+1.75 | 39+2.83 | 38.72 +1.29 | *p* = 0.932; OR = 1.027[0.583,1.758] |
| Baby birth weight (gram) |  | 3446.4 + 826.026 | 3440+84.85 | 3410.25 + 558.3 | *p* = 0.467; OR = 0.999[0.995, 1.001] |
| NICU or SCN admission | No | 7 | 1 | 12 | *p* = 0.558; OR = 2.25[0.256,31.272] |
|  | Yes | 7 | 1 | 10 |  |
|  | Missing | 1 |  | 2 |  |
| Apgar score - 1 mins |  | 8.23+1.17 | 5+1.41 | 7.85+1.79 | *p* = 0.437; OR = 0.333[0.023,3.0] |
| Apgar score - 5 mins |  | 8.88+0.51 | 9+0 | 9.05+0.83 | *Not applicable* |

OR: odds ratio; LSCS: lower segment caesarean section; GDM: Gestational Diabetes Mellitus; NICU: neonatal intensive care unit; SCN: special care nursery; significance level was set at 0.05; participants with more than three missing outcomes were not included in this analysis.

**Supplementary Table 2.** Sensitivity analysis in the subgroup (Marital Status = No)

|  |  | Control | Clozapine | Quetiapine |  |
| --- | --- | --- | --- | --- | --- |
| Birth Mode |  |  |  |  | *Not applicable* |
|  | Live birth | 9 | 8 | 20 |  |
|  | Miscarriage | 0 | 1 | 0 |  |
|  | Missing | 0 | 0 | 3 |  |
| Delivery Model |  |  |  |  | *P = 0.963; OR = 0.992[0.747,1.328]* |
|  | Vaginal | 4 | 5 | 9 |  |
|  | Vaginal/Forceps | 1 | 1 | 0 |  |
|  | Vaginal/Venteuse | 0 | 0 | 1 |  |
|  | Vaginal/Venteuse and Forceps | 0 | 0 | 0 |  |
|  | Emergency LSCS | 2 | 2 | 3 |  |
|  | Elective LSCS | 2 | 1 | 6 |  |
|  | Missing | 0 | 0 | 4 |  |
| Weight gain overall (kilogram) |  | 17 + 9.10 | 11.22+5.93 | 16.58+12.80 | *p* = 0.608; OR = 0.981[0.921,1.042] |
| GDM |  |  |  |  | *p* = 0.591; OR = 0.980[0.918,1.04] |
|  | **Without family history** |  |  |  |  |
|  | No | 2 | 1 | 9 |  |
|  | Yes | 0 | 1 | 1 |  |
|  | Missing | 0 | 0 | 2 |  |
|  | **With family history** |  |  |  |  |
|  | No | 6 | 1 | 8 |  |
|  | Yes | 1 | 6 | 3 |  |
|  | Missing | 0 | 0 | 0 |  |
| Fetal distress |  |  |  |  | *p* = 0.995 |
|  | No | 7 | 9 | 22 |  |
|  | Yes | 2 | 0 | 0 |  |
|  | Missing |  |  | 1 |  |
| Gestational age (weeks) |  | 36.84 + 4.36 | 37.33 + 1.66 | 39.13 + 1.80 | *p* = 0.130; OR = 1.235[0.99,1.601] |
| Baby birth weight (gram) |  | 2928.22 + 1032.54 | 3118.89 + 517.1 | 3582.9 + 501.25 | *p* = 0.08; OR = 1.001[1,1.002] |
| NICU or SCN admission | No | 6 | 2 | 11 | *p* = 0.297; OR = 2.308[0.64,9.433] |
|  | Yes | 3 | 7 | 8 |  |
|  | Missing |  |  | 4 |  |
| Apgar score - 1 mins |  | 8.44 + 0.53 | 8.67 + 0.52 | 8.22+1.22 | *p* = 0.765; OR = 0.877[0.378,1.71] |
| Apgar score - 5 mins |  | 9+0 | 9+0 | 8.92 + 1.22 | *p* = 0.549; OR = 0.299[0.003,4.386] |

OR: odds ratio; LSCS: lower segment caesarean section; GDM: Gestational Diabetes Mellitus; NICU: neonatal intensive care unit; SCN: special care nursery; significance level was set at 0.05; participants with more than three missing outcomes were not included in this analysis.

**Supplementary Table 3.** Sensitivity analysis in the subgroup (Antidepressant 1st trimester = 0)

|  |  | Control | Clozapine | Quetiapine |  |  |
| --- | --- | --- | --- | --- | --- | --- |
| Birth Mode |  |  |  |  | *p* = 0.995 | |
|  | Live birth | 18 | 4 | 21 |  | |
|  | Miscarriage | 0 | 0 | 0 |  | |
|  | Missing | 0 | 0 | 1 |  | |
| Delivery Model |  |  |  |  | *p* = 0.679; OR = 0.915 [0.625, 1.29] | |
|  | Vaginal | 8 | 3 | 9 |  | |
|  | Vaginal/Forceps | 3 | 1 | 0 |  | |
|  | Vaginal/Venteuse | 0 | 0 | 1 |  | |
|  | Vaginal/Venteuse and forceps | 1 | 0 | 0 |  | |
|  | Emergency LSCS | 4 | 0 | 2 |  | |
|  | Elective LSCS | 1 | 0 | 9 |  | |
|  | Missing | 1 | 0 | 1 |  | |
| Weight gain overall (kilogram) |  | 18.13+9.87 | 8.75+7.80 | 18.64+7.82 | *p* = 0.413; OR = 0.958[ [0.873,1.04] | |
| GDM |  |  |  |  | *p* = 0.994 | |
|  | **Without family history** |  |  |  |  | |
|  | No | 9 | 1 | 9 |  | |
|  | Yes | 1 | 0 | 0 |  | |
|  | Missing | 0 | 0 | 1 |  | |
|  | **With family history** |  |  |  |  | |
|  | No | 7 | 2 | 8 |  | |
|  | Yes | 1 | 1 | 4 |  | |
| Fetal distress |  |  |  |  |  | |
|  | No | 15 | 4 | 22 | *p* = 0.995 | |
|  | Yes | 3 | 0 | 0 |  | |
| Gestational age (weeks) |  | 38.05+3.33 | 39+1.63 | 39.17+1.73 | *p* = 0.728; OR = 1.013[0.684, 1.773] | |
| Baby birth weight (gram) |  | 3276.556+1045.961 | 3585+222.94 | 3459.05+572.52 | *p* = 0.413; OR = 0.958[0.873,1.04] | |
| NICU or SCN admission | No | 10 | 2 | 14 | *p* = 0.199; OR = 3.4[0.747,19.019] | |
|  | Yes | 7 | 2 | 7 |  | |
|  | Missing | 1 |  | 1 |  | |
| Apgar score - 1 mins |  | 8.41+0.87 | 6.33+2.52 | 8.19+1.6 | *p* = 1.000; OR = 1 [0.479, 1.826] | |
| Apgar score - 5 mins |  | 9.03+0.28 | 9+0 | 9+0.55 | *p* = 0.252; OR = 2.361[0.688, 8.979] | |

OR: odds ratio; LSCS: lower segment caesarean section; GDM: Gestational Diabetes Mellitus; NICU: neonatal intensive care unit; SCN: special care nursery; significance level was set at 0.05; participants without information of antidepressant use in the 1^st^ trimester were not included in this analysis.

**Supplementary Table 4.** Sensitivity analysis in the subgroup (Antidepressant 1st trimester = 1)

|  |  | Control | Clozapine | Quetiapine |  |
| --- | --- | --- | --- | --- | --- |
| Birth Mode |  |  |  |  | Not applicable |
|  | Live birth | 6 | 6 | 22 |  |
|  | Miscarriage | 0 | 1 | 0 |  |
|  | Missing | 0 | 0 | 3 |  |
| Delivery Model |  |  |  |  | *p* = 0.274; OR = 1.175 [0.926, 1.511] |
|  | Vaginal | 2 | 3 | 7 |  |
|  | Vaginal/Forceps | 0 | 1 | 0 |  |
|  | Vaginal/Venteuse | 0 | 0 | 1 |  |
|  | Vaginal/Venteuse and Forceps | 0 | 0 | 0 |  |
|  | Emergency LSCS | 2 | 2 | 6 |  |
|  | Elective LSCS | 2 | 1 | 6 |  |
|  | Missing | 0 | 0 | 5 |  |
| Weight gain overall (kilogram) |  | 18.13+9.87 | 8.75+7.80 | 18.64+7.82 | *p* = 0.718; OR = 0.988[0.936, 1.043] |
| GDM |  |  |  |  | *p* = 0.513; OR = 1.837[0.423, 9.986] |
|  | **Without family history** |  |  |  |  |
|  | No | 2 | 1 | 9 |  |
|  | Yes | 0 | 1 | 1 |  |
|  | Missing | 0 | 0 | 2 |  |
|  | **With family history** |  |  |  |  |
|  | No | 4 | 0 | 7 |  |
|  | Yes | 0 | 5 | 6 |  |
| Fetal distress |  |  |  |  | *p* = 0.994 |
|  | No | 6 | 7 | 22 |  |
|  | Yes | 0 | 0 | 1 |  |
|  | Missing | 0 | 0 | 1 |  |
| Gestational age (weeks) |  | 37.93+1.90 | 36.86+1.57 | 38.65+1.31 | *p* = 0.188; OR = 1.204[0.975,1.577] |
| Baby birth weight (gram) |  | 3178.667+435.95 | 2944.286+432.25 | 3558.05+493.17 | *p* = 0.404; OR = 1[1, 1.001] |
| NICU or SCN admission | No | 4 | 1 | 9 | *p* = 0.577; OR = 0.703[0.246,1.994] |
|  | Yes | 2 | 6 | 11 |  |
|  | Missing |  |  | 5 |  |
| Apgar score - 1 mins |  | 8+1.22 | 8.6+0.55 | 7.82+1.47 | *p* = 0.346; OR = 0.779[0.465,1.148] |
| Apgar score - 5 mins |  | 8.6+0.55 | 9+0 | 8.97+0.76 | *p* = 0.826; OR = 0.844[0.202, 3.035] |

OR: odds ratio; LSCS: lower segment caesarean section; GDM: Gestational Diabetes Mellitus; NICU: neonatal intensive care unit; SCN: special care nursery; significance level was set at 0.05; participants without information of antidepressant use in the 1^st^ trimester were not included in this analysis.
